# Manipulation of the Rice L-Galactose Pathway: Evaluation of the Effects of Transgene Overexpression on Ascorbate Accumulation and Abiotic Stress Tolerance

**DOI:** 10.1371/journal.pone.0125870

**Published:** 2015-05-04

**Authors:** Gui-Yun Zhang, Ru-Ru Liu, Chang-Quan Zhang, Ke-Xuan Tang, Ming-Fa Sun, Guo-Hong Yan, Qiao-Quan Liu

**Affiliations:** 1 Key Laboratory of Crop Genetics and Physiology of Jiangsu Province, Co-Innovation Center for Modern Production Technology of Grain Crops, Yangzhou University, Yangzhou 225009, Jiangsu, China; 2 Agricultural Science Institute of Coastal Region of Jiangsu, Yancheng 224002, Jiangsu, China; 3 School of Agriculture and Biology, Shanghai Jiao Tong University, Shanghai 200240, China; Institute of Genetics and Developmental Biology, Chinese Academy of Sciences, CHINA

## Abstract

Ascorbic acid (AsA) is the most abundant water-soluble antioxidant in plants, and it plays a crucial role in plant growth, development and abiotic stress tolerance. In the present study, six key *Arabidopsis* or rapeseed genes involved in AsA biosynthesis were constitutively overexpressed in an elite *Japonica* rice cultivar. These genes encoded the GDP-mannose pyrophosphorylase (GMP), GDP-mannose-3',5'-epimerase (GME), GDP-L-galactose phosphorylase (GGP), L-galactose-1-phosphate phosphatase (GPP), L-galactose dehydrogenase (GDH), and L-galactono-1,4-lactone dehydrogenase (GalLDH). The effects of transgene expression on rice leaf AsA accumulation were carefully evaluated. In homozygous transgenic seedlings, *AtGGP* transgenic lines had the highest AsA contents (2.55-fold greater than the empty vector transgenic control), followed by the *AtGME* and *AtGDH* transgenic lines. Moreover, with the exception of the *AtGPP* lines, the increased AsA content also provoked an increase in the redox state (AsA/DHA ratio). To evaluate salt tolerance, *AtGGP* and *AtGME* transgenic seedlings were exposed to salt stress for one week. The relative plant height, root length and fresh weight growth rates were significantly higher for the transgenic lines compared with the control plants. Altogether, our results suggest that GGP may be a key rate-limiting step in rice AsA biosynthesis, and the plants with elevated AsA contents demonstrated enhanced tolerance for salt stress.

## Introduction

L-ascorbic acid (AsA), also known by its popular name vitamin C, is the most abundant water-soluble antioxidant in plants. It protects plants from oxidative damage by scavenging the free radicals and reactive oxygen species generated by photosynthesis, oxidative metabolism, and environmental stresses [1]. AsA has been shown to play a critical role in plant growth and development, as well as in tolerance of abiotic stresses such as drought [2], salinity [3,4], ozone [5], and high light intensity [6]. Additionally, AsA is a cofactor for many enzymes that are involved in multiple processes, including flavonoid and phytohormone biosynthesis as well as the xanthophyll cycle [7, 8].

**Fig 1:**
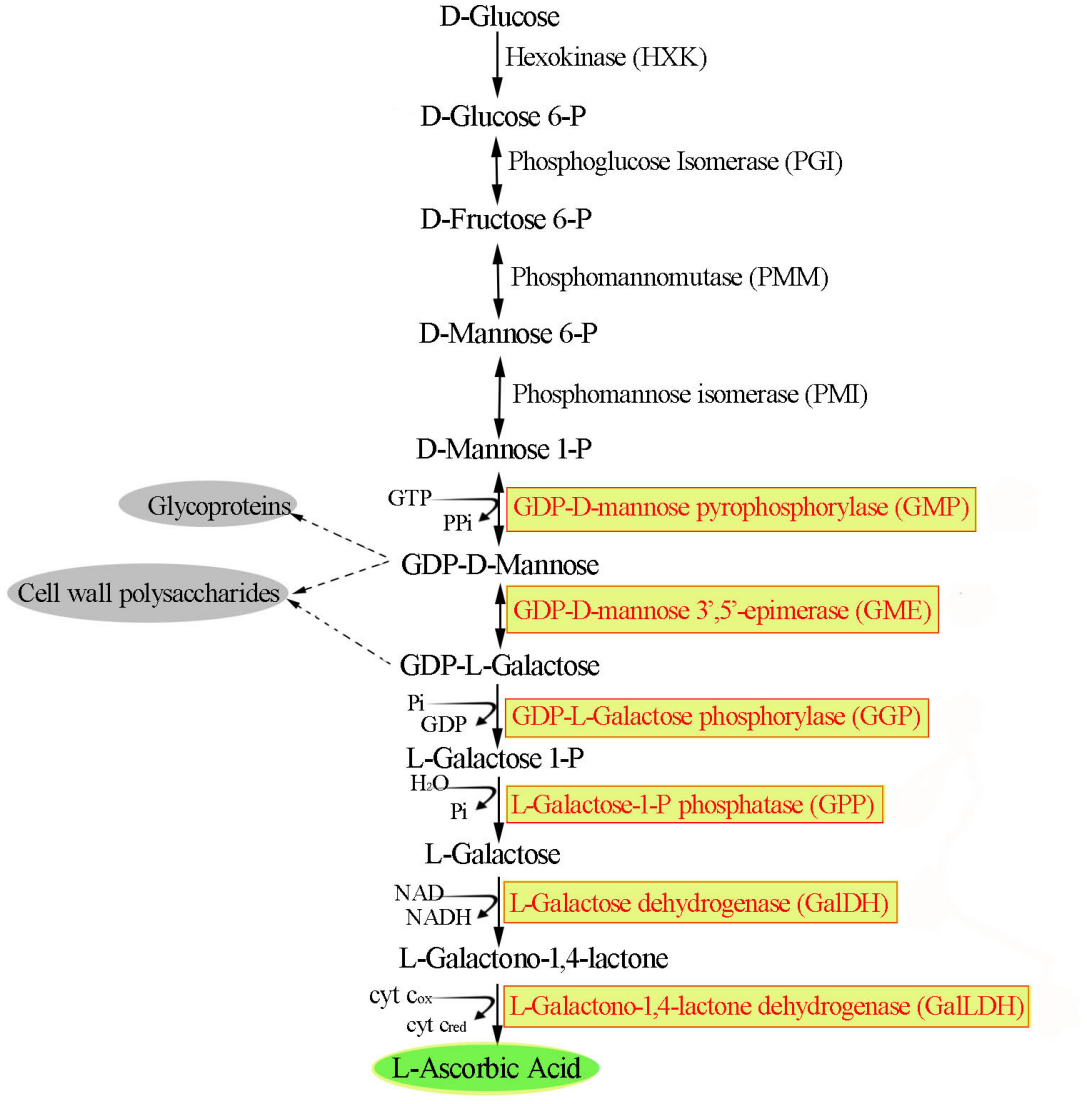
The L-galactose pathway in higher plants [14].

To date, four alternative AsA biosynthesis pathways have been proposed in plants: the L-galactose [9], galacturonate [10,11], glucose [12] and myo-inositol pathways [13]. Among these pathways, the L-galactose pathway might have an important role in cereal AsA biosynthesis (Fig 1) [14]. For example, when rice seedlings were supplied with L-galactose and L-galactono-1,4-lactone, shoot AsA contents increased up to 5- and 4-fold, respectively; in contrast, for D-galacturonic acid, myo-inositol, D-glucuronolactone and L-guluno-1,4-lactone, AsA levels were similar to those of the untreated control plants [15]. At least ten enzymes in the L-galactose pathway are involved in converting D-glucose to AsA through the formation of GDP-D-mannose, GDP-L-galactose, L-galactose-1-P, L-galactose, and L-galactono-1,4-lactone intermediates (Fig 1).

Most of the genes involved in this pathway, e.g., GDP-mannose pyrophosphorylase (GMP) [16,17], GDP-mannose-3',5'-epimerase (GME) [3], GDP-L-galactose phosphorylase (GGP) [18,19], L-galactose-1-phosphate phosphatase (GPP) [20], L-galactose dehydrogenase (GDH) [21], and L-galactono-1,4-lactone dehydrogenase (GalLDH) [22,23], have been cloned, and several researchers have attempted to regulate AsA content by overexpressing them in transgenic plants. However, these studies each only focused on one gene, and few works have examined the effects of transgenic regulation of all of the above genes in one experiment, especially in rice, one of the most important crops consumed worldwide.

Feeding with AsA precursors during rice growth led to an increase in leaf AsA content, shedding light onto the development of cultivars with enhanced tolerance for key environmental stresses [24–26]. In contrast, the ND6172 rice mutant had approximately 20–30% lower AsA levels and exhibited negative effects on agronomic traits at ozone levels exceeding the current ambient concentrations [27]. Moreover, reducing *GalLDH* expression in rice was reported to increase lipid peroxidation, but also decrease foliar AsA levels [22]. These results revealed that the AsA content of rice leaves is a sensitive characteristic that might be consistent with stress tolerance. Furthermore, the AsA redox state, i.e., the ratio between ascorbate and dehydroascorbate (AsA/DHA), also plays important role in plant senescence, defense, and stress response [28–30]. The AsA/DHA ratio may depend on the plant species; in general, it is approximately 1.0 in 4-week-old rice seedlings [31, 32].

In the present study, the expression levels of genes involved in the rice L-galactose pathway were compared by quantitative RT-PCR. Six L-galactose pathway genes from *Arabidopsis* or rapeseed, including *GMP*, *GME*, *GGP*, *GPP*, *GDH* and *GalLDH* (Fig 1), were then overexpressed in rice and their effects on rice seedling AsA accumulation were carefully compared. This results of this analysis showed that *GGP* overexpression had the best AsA accumulation, followed by *GME* and *GDH*, and the abiotic stress tolerances of *GGP* and *GME* transgenic rice plants were subsequently evaluated.

## Materials and Methods

### Plant materials

The *Japonica* rice variety Nipponbare was used for quantitative expression analysis of the endogenous L-galactose pathway genes. Another elite *Japonica* cultivar, Wuyujing 3 (WY3), was used for generation of transgenic lines. All rice plants were grown in the field at the experimental farm of Yangzhou University (32°39’N, 119°42’E) under normal culture conditions. Developing endosperm, stems, sheaths and old leaves were sampled and collected at 20 days, and young leaves and roots were harvested from 4-week-old seedlings. The collected samples were immediately frozen in liquid nitrogen and stored at −80°C.

### Transgenic vector construction

**Fig 2:**
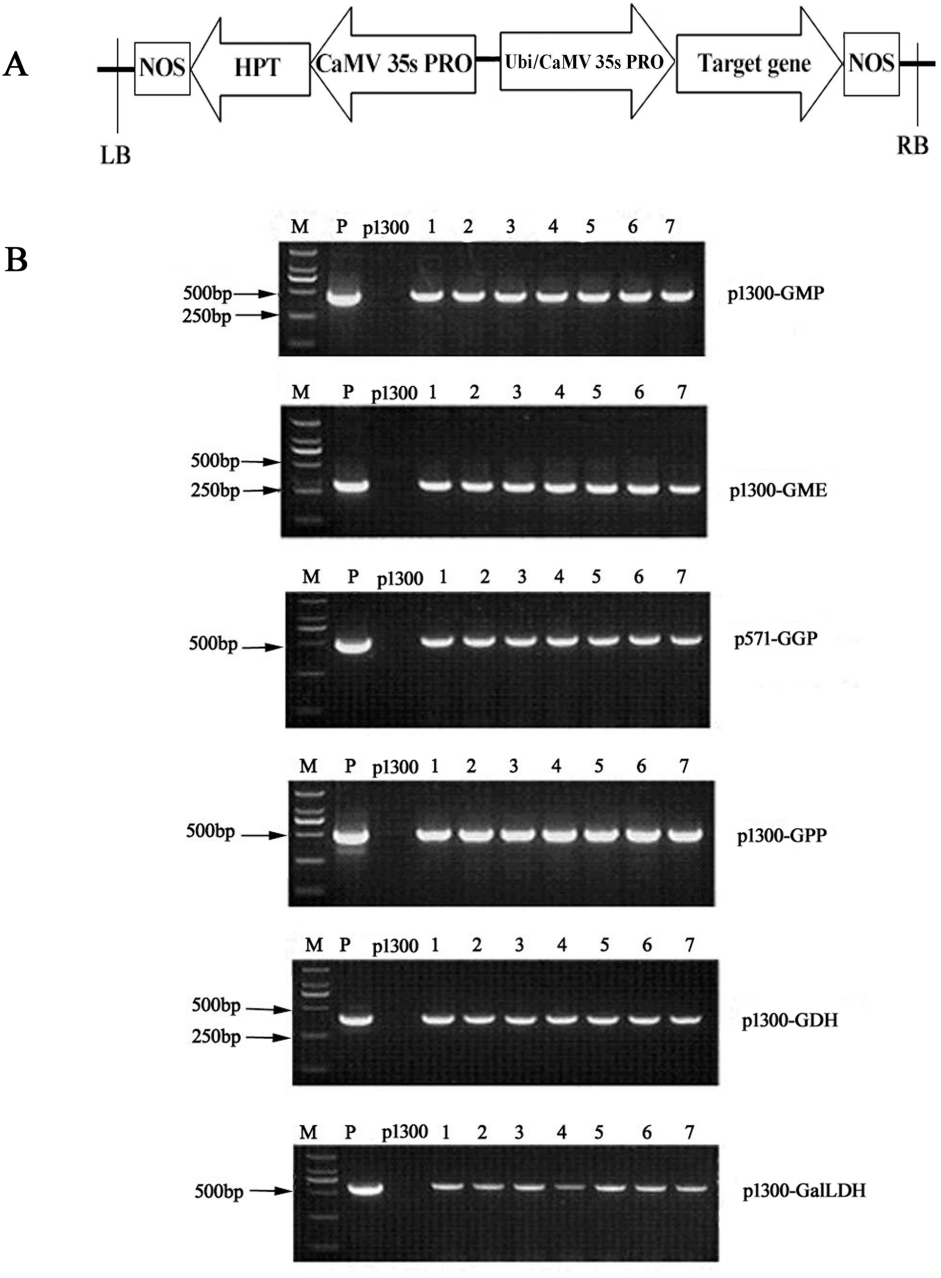
Rice transformation constructs (A) and PCR amplification of transformed genes (B). A. Schematic representation of the transformation vectors used in this study. *CaMV 35S pro*, cauliflower mosaic virus 35S promoter; *Ubi pro*, maize ubiquitin promoter; *NOS*, nopaline synthase gene terminator; *HPT*, hygromycin resistance gene; LB, left border; RB, right border. B. PCR analysis of transgenic and control lines using specific primers for amplification of each transgene. Lanes 1–7, transgenic plants (T_0_ generation); P, positive control; M, DL2000 DNA marker (TAKARA, Dalian, China).

*Arabidopsis AtGGP*, *AtGMP*, *AtGME*, *AtGPP*, *AtGDH* and rapeseed *BoGalLDH* cDNAs (GenBank accession numbers AY093138, AF076484, NM_122767, NM_001035549, AJ417563, and Z97060, respectively) were previously cloned by RT-PCR [20]. To construct the transgenic vectors, the cDNAs were amplified to add suitable restriction enzymatic sites using the primers listed in S1 Table. The amplified cDNAs were then inserted into the pMD18-T vector (TAKARA, Dalian, China) and confirmed by sequence analysis. The full-length cDNAs, regulated by the maize *Ubi* or *CaMV 35s* promoters and NOS terminator, were cloned into the binary pCAMBIA1300 vector (www.cambia.com.au). With the exception of *AtGGP*, which was driven by the *CaMV 35s* promoter, the target genes were regulated by the maize *Ubi* promoter. The resulting binary vectors (Fig 2) containing the target genes and the corresponding empty pCAMBIA1300 control vector were transformed into *Agrobacterium tumefacien* strain EHA105 and subsequently used for rice transformation.

### Rice transformation and confirmation

Calli derived from either immature or mature WY3 rice cultivar embryos were used as explants for *Agrobacterium*-mediated transformation. Tissue culture, transformation, selection and regeneration of transgenic plants were performed as described by Liu et al. [33]. Genomic DNA was extracted from leaves using the CTAB method [34], and PCR analysis was performed using transgene-specific primer sets (S1 Table) to detect the transgenes.

### Quantitative RT-PCR

Total RNA was isolated as previously described [35]. A 1 μg sample of each RNA preparation was used for reverse transcription using the SuperScript First-Strand Synthesis System (Invitrogen, Carlsbad, CA) and oligo(dT)_18_ according to the manufacturer’s instructions. Aliquots of cDNA were used in 25 μl real-time PCR assay reactions using the SYBR Premix ExTaq kit (TaKaRa, Tokyo, Japan). The results were normalized against rice *actin* mRNA. The sequences of the real-time PCR assay primers are listed in S1 Table.

### AsA and DHA measurements

AsA was extracted according to the method described by Zhou et al. [20]. Briefly, 50 mg of leaves from 4-week-old seedlings were harvested at 6 pm, ground in liquid nitrogen, mixed with 1 ml 0.1% oxalic acid in dim light, and placed on ice for 1 h. The samples were centrifuged at 10 000 g for 10 min and the supernatants were then pooled and filtered for measurement. For AsA assays measuring the reduced + oxidized forms, 25 μL (25 mmol L^-1^) DTT and 25 μL (320 mmol L^-1^) K_2_HPO_4_ were added to 500 μL supernatant, incubated for 15 minutes at room temperature, centrifuged, and then filtered for measurements. The amounts of DHA were determined as the difference between the two assays. Standard L-AsA solutions (1 mg ml^-1^) (Sigma, St. Louis, MO, USA) were freshly prepared prior to use. L-AsA HPLC analysis was performed using a C-18 reversed phase column (5 μm, 150×4.6 mm). Samples (10 μL) were injected using a Waters 2695 autosampler. The solvent system was 0.1% oxalic acid at a 1.0 mL min^-1^ flow rate, and the samples were analyzed with a UV detector at 245 nm.

### Salt stress treatment

Germinated seeds from transgenic and control lines were placed in the 96-well PCR plate (without bottom) and pre-grown in modified Hoagland’s solution (S2 Table). The culture was under a light intensity of about 700 μmol photons m^−2^ s^−1^, 75% humidity and a 16/8-h light/dark photoperiod at 25°C for three weeks. Seedlings were then cultured in solutions containing 100 mM NaCl for one week. The solutions were renewed everyday, and three replications were used in salt stress evaluation experiments. The plant heights, root lengths and fresh weights were measured before and after salt stress, and the relative plant height, root length and fresh weight growth rates were calculated.

### Relative membrane ion leakage analysis

The oxidative stress tolerance of the plants was assessed by ion leakage analysis as described by Wang et al. [36]. Ten leaf discs of 6 mm diameter were placed in a cuvette containing 20 mL distilled water, vacuumized for 30 min, and then surged for 10 h at room temperature to measure the initial electronic conductance (S1) using an osmometer (DDS-11A, SuoShen, Shanghai, China). The cuvette was heated in boiling water for 30 min and cooled to room temperature to determine the final electronic conductance (S2). The relative membrane ion leakage (%) was defined as 100× (S1/S2).

### Statistical analysis

The data were analyzed using a statistical software package (SPSS v16.0). One-way analysis of variance (ANOVA) was used to compare the differences between the transgenic and control lines, followed by Tukey’s post hoc test. A p-value below 0.05 was considered to be statistically significant.

## Results

### Enriched expression of the L-galactose pathway in young rice leaves

**Fig 3:**
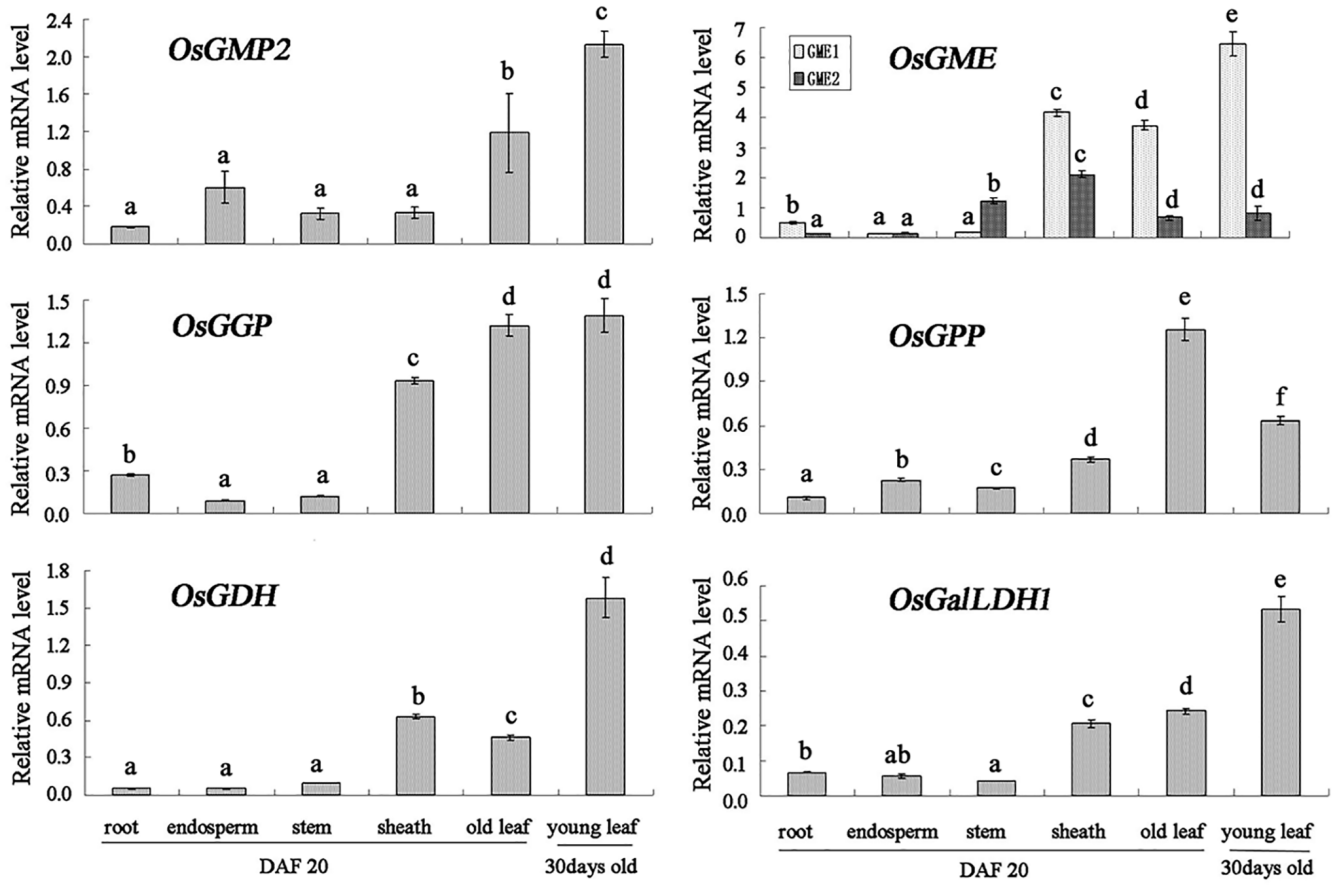
Expression of ascorbate biosynthetic genes in rice roots, endosperm, stems, sheaths, old and young leaves. All tissues except young leaves were taken from DAF20 (20 days after flowering) plants. The young leaves were taken from 30 day-old plants. Data obtained by real-time RT-PCR were normalized using *actin* mRNA expression. Data shown are the average of three measurements with standard errors. Different letters indicate statistically significant differences at P<0.05 (ANOVA, Tukey post-host). The GeneBank accession numbers for *OsGMP2*, *OsGME1*, *OsGME2*, *OsGGP*, *OsGPP*, *OsGDH*, and *OsGalLDH1* are AK122126, AK069385, AK102348, AK071188, AK071149, AK067039, and AK241565, respectively.

The rice genome contains only one copy each of the *OsPMM*, *OsGGP* and *OsGPP* genes, while there are two copies of the *OsGME* genes (*OsGME*1 and *OsGME*2) and *OsGalLDH* (*OsGalLDH1* and *OsGalLDH2*) and three copies of *OsGMP* genes (*OsGMP1*, *OsGMP2* and *OsGMP3*) [37]. To increase our understanding of rice AsA biosynthesis, we first performed expression profiling of the genes involved in the final six L-galactose pathway steps in various tissues. As there is high identity (more than 90%) among the coding sequences of three *OsGMP* genes, and also very high identity (98%) between *OsGalLDH1* and *OsGalLDH2*, the expression analysis was only carried out by using the primer according to the *OsGMP2* or *OsGalLDH1*, which might represent the whole of three *OsGMP* genes or two *OsGalLDH* genes, respectively. As shown in Fig 3, this analysis demonstrated expression of all tested genes in all tissues; however, their transcription levels were quite low in root, stem and developing endosperm samples compared with their levels in leaves. Moreover, their expression in young leaves from 4-week-old seedlings were much greater than in old leaves from plants 20 days after flowering, implying that the expression of L-galactose pathway was enriched in young rice leaves.

### Generation and confirmation of homozygous transgenic rice

Expression cassettes (Fig 2A) containing the target gene or empty vector were transferred into WY3 *Japonica* rice by *Agrobacterium*-mediated transformation. More than 20 independent transformants were regenerated for each construct. All T_0_ transgenic rice plants were confirmed by PCR using primers specific to each target gene (Fig 2B). The results from field studies showed that there was no significant difference of the phenotype such as plant height, tiller number, flower time and seed production between the transgenic plants and their wild type under normal field condition. For each construct, at least three independent lines with homozygous and high-levels of transgene expression were chosen for further analysis. The transgenic plants derived from the empty vector p1300 (empty control) were also selected for further testing.

### Transgene expression in rice leaves

**Fig 4:**
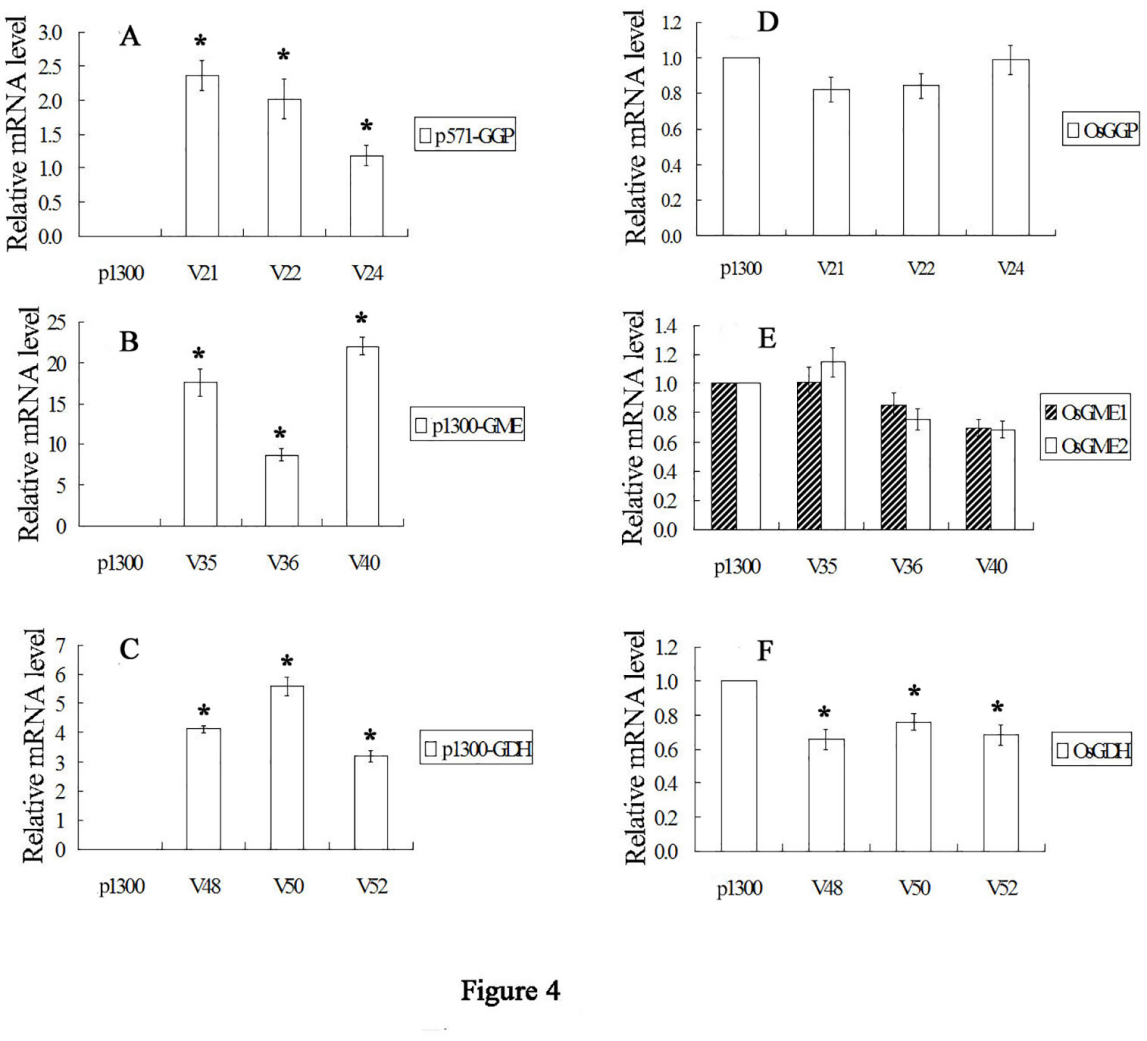
Relative gene expression in *AtGGP*, *AtGME* and *AtGDH* transgenic lines. A, *AtGGP*; B, *AtGME*; C, *AtGDH*; D, *OsGGP*; E, *OsGME1* and *OsGME2*; F, *OsGDH*. A-C, data obtained by real-time RT-PCR were normalized using *actin* mRNA expression. E-F, data obtained by real-time RT-RCR were normalized using *actin* expression and are expressed as a percentage of the control. *Asterisk* indicates values that are significantly different from those of empty control plants (Tukey’s test, *P*<0.05).

Transgene expression was detected by real-time RT-PCR, and the results of this analysis showed that the foreign transgenes could be expressed at high levels in rice leaves (Fig 4A–4C). The expression of endogenous genes in transgenic rice plants was also analyzed and compared with the empty control (Fig 4D–4F). In *AtGDH* transgenic rice leaves, endogenous *OsGDH* expression was slightly lower than empty control (Fig 4F); however, the expression levels of endogenous genes related to AsA biosynthesis in the other transgenic plants were quite similar to those of the empty control (Fig 4D and 4E). The results of this analysis suggested that the transgenes had limited or no effects on L-galactose pathway gene expression.

### Effects of exogenous gene overexpression on AsA accumulation

**Fig 5:**
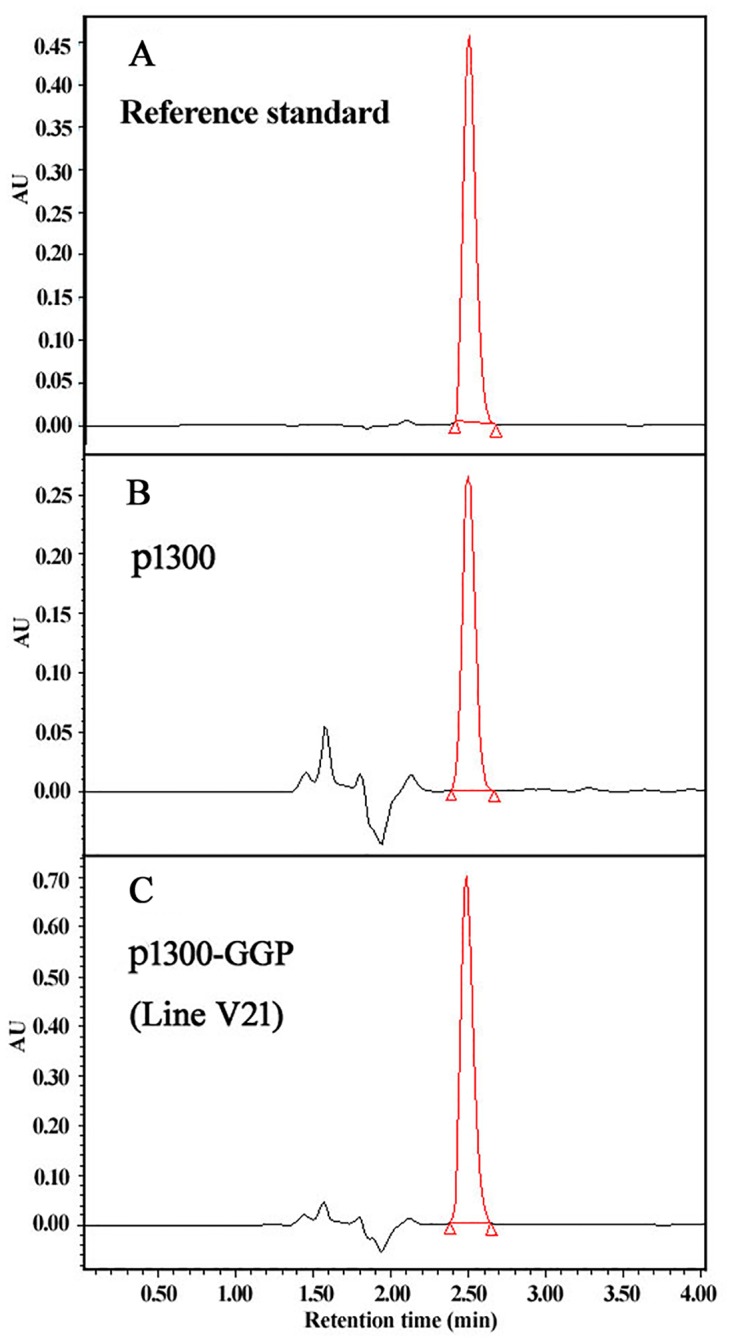
HPLC chromatograms of AsA standards (A), and extracts from the leaves of empty control (B) and p571-GGP-derived transgenic rice (C).

**Fig 6:**
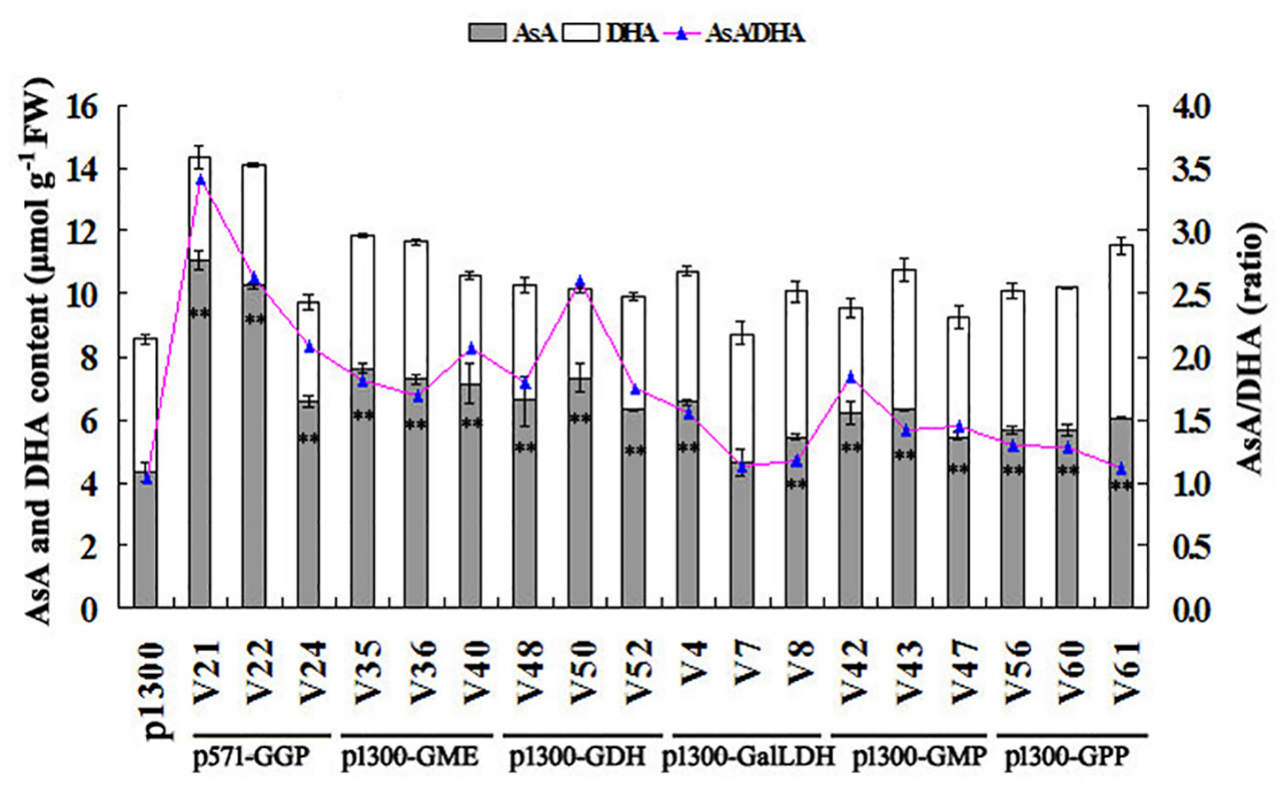
AsA and DHA contents and the ratio of AsA to DHA in each transgenic line. Data are the averages of thirty plants ± standard deviation. Double asterisk indicates values of AsA content are significantly different from those of empty control plants (Tukey’s test, *P*<0.01).

To analyze and compare AsA levels among different transgenic rice plants, T_6_ generation homozygous transgenic lines and the empty p1300 control were grown simultaneously in the same field under normal culture conditions. The young, fully expanded fresh leaves of 30 day-old plants were collected and used for HPLC measurement of AsA and DHA levels. We used 0.1% oxalic acid as the extraction buffer and found that it provided high AsA stabilization and minimal interaction with the chromatographic system (Fig 5). Compared with the p1300 empty control, AsA levels were significantly increased (up to 2.55-fold) for all six target transgenic lines (Fig 6). Among the transgenic lines, the p571-GGP lines showed the best AsA accumulation, followed by the p1300-GME and p1300-GDH transgenic lines.

### Effects of exogenous gene overexpression on the ascorbate redox state

As shown in Fig 6, the DHA levels were not significantly altered in the leaves of transgenic rice overexpressing the target genes. As the AsA levels increased significantly in these transgenic leaves, the redox state (AsA/DHA ratio) also increased in the transgenic lines, with the exception of the p1300-GPP lines. The ratio of AsA to DHA was approximately 1.04 in the empty control (p1300) plants, while it was 3.42, 2.64 and 2.08 in the leaves of p1300-GGP transgenic lines V21, V22 and V24, respectively (Fig 6).

### Increased AsA content leads to enhanced salt stress tolerance

**Fig 7:**
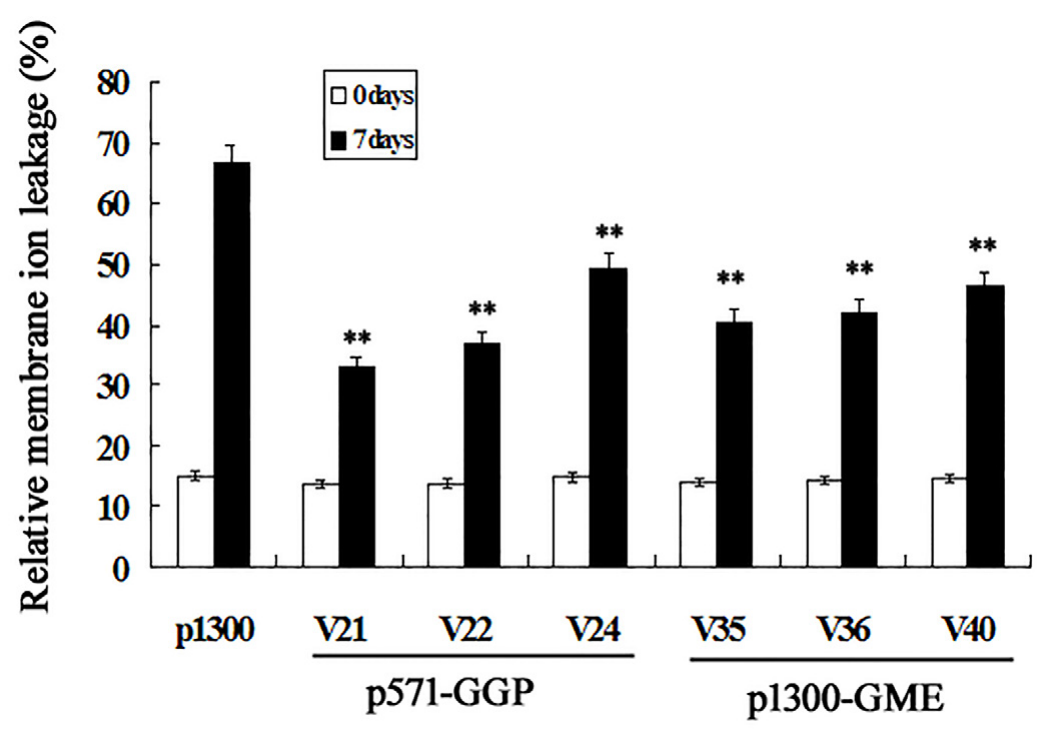
Relative membrane ion leakage in the leaves before and after salt stress treatment. The data presented are the mean values of twelve plants ± standard deviation. *Asterisk* indicates values that are significantly different from those of empty control plants (Tukey’s test, *P*<0.05).

To investigate the effects of increased AsA content on transgenic rice salt tolerance, p1300-GGP and p1300-GME transgenic rice plants which were the two best in AsA accumulation were selected for stress tolerance testing. The 3-week old rice seedlings were transferred to Hoagland’s solution supplemented with 100 mM NaCl for one week. The plant heights, root lengths and fresh weights were measured before and after salt stress, and used to calculate the relative growth rates (Table 1). This analysis showed that the relative plant height and root length growth rates in most of the p1300-GME or p571-GGP transgenic lines tested were significantly greater (*p*<0.01) than those of the p1300 control plants. The fresh weights of the p1300 control plants were reduced after salt treatment due to wilting under salt stress; in contrast, the extent of wilting was reduced for the p571-GGP and p1300-GME transgenic lines. Consequently, the relative fresh weight growth rates of the p571-GGP and p1300-GME transgenic lines were significantly higher (*p*<0.01) than those of the p1300 control plants (Table 1). The ion leakage was measured to reflect the level of cellular damage after salt treatment. As shown in Fig 7, relative membrane ion leakage was not significantly different between wild-type and transgenic plants under normal conditions. However, after salt treatment, the relative membrane ion leakage of wild-type plants increased up to 67%, significantly higher (*p*<0.01) than that of transgenic plants. The results indicated that overexpression of *AtGGP* or *AtGME* could significantly improve rice seedling salt tolerance.

**Table 1:** The relative growth rate of plant height, root length and fresh weight growth rates of transgenic lines transferred to 100 mM NaCl for one week.

Transgene	Line	Plant height (cm) before stress	Plant height (cm) after stress	The relative growth rate of plant height	Root length (cm) before stress	Root length (cm) after stress	The relative growth rate of root length	Fresh weight (mg/plant) before stress	Fresh weight (mg/plant) after stress	The relative growth rate of fresh weight
Empty	p1300	15.87±0.21	16.17±0.32	+0.02	9.57±0.31	10.73±0.25	+0.12	206.33±7.37	185.33±5.13	-0.10
*AtGGP*	V21	16.06±0.25	16.40±0.43	+0.02	10.28±0.21	11.98±0.15**	+0.17**	218.38±9.76	251.01±4.58**	+0.15**
	V22	16.02±0.14	16.78±0.65*	+0.05**	10.17±0.15	11.77±0.32**	+0.15*	214.34±6.31	286.49±4.16**	+0.34**
	V24	15.47±0.15	16.80±0.26**	+0.09**	10.13±0.15	11.37±0.21	+0.12	208.21±8.79	241.67±7.81**	+0.16**
*AtGME*	V35	15.41±0.21	16.94±0.21**	+0.10**	9.67±0.40	11.62±0.13**	+0.20**	209.46±7.10	235.08±6.03**	+0.12**
	V36	15.20±0.36	17.18±0.23**	+0.13**	9.54±0.17	11.53±0.26*	+0.21**	202.33±8.22	247.29±7.21**	+0.22**
	V40	15.16±0.37	17.43±0.26**	+0.15**	9.23±0.46	11.05±0.32	+0.20**	193.37±5.03	207.67±5.51*	+0.07**

The data is presented as mean ± SD of at least twelve plants.

* and ** represent significant differences (Tukey’s test) at *p*≤0.05 and *p*≤0.01, respectively.

## Discussion

In the last decade, great progress has been made in metabolic engineering of the L-galactose pathway; however, it is difficult to systematically compare the contributions of L-galactose pathway genes to AsA accumulation due to the differences in transgenes and plant species across studies.

In this study, six genes involved in the L-galactose pathway were overexpressed in rice. Our analysis showed that the p571-GGP transgenic lines had the highest AsA levels, with a 2.55-fold increase compared with the empty vector control; similar results were reported by Zhou et al. [20]. Transgenic tobacco plants overexpressing kiwifruit *GGP* showed an approximately 3-fold increase in AsA levels [38]. In 2014, Wang et al. [18] reported that overexpression of tomato *GGP* in tobacco led to a significant increase in the AsA content and AsA/DHA ratio. Therefore, we can conclude that GGP may be a key rate-limiting step in plant AsA biosynthesis, especially in *Arabidopsis*, tobacco and rice.

The reactions catalyzed by GMP and GME are located in the upper steps of the end of AsA biosynthesis, and they are also important for cell wall formation and membrane protein modification. Overexpression of tomato *GMP* in tobacco resulted in a 2-4-fold increase in AsA and improved temperature stress tolerance [17]. Expression of *GMP* increased AsA levels in tomatoes up to 70% in leaves, 50% in green fruit and 35% in red fruit [39]. In this report, p1300-GMP transgenic lines exhibited a half-fold increase in AsA levels compared with the controls; therefore, GMP does not appear to exert a major metabolic function during rice AsA biosynthesis. Overexpression of both *SlGME1* and *SlGME2* resulted in a 1.4-fold increase in the AsA contents of tomato leaves and fruits, and improved abiotic stress tolerance [3]. It was reported that the OsGME kinetic parameters seemed to be relatively low for rapid AsA biosynthesis [40]. Overexpression of *AtGME* in rice showed the next best enhancement of AsA levels. GME catalyzes the double epimerization of the D-mannose moiety at the 3' and 5' carbons to form L-galactose, and the reaction equilibrium is highly biased against AsA biosynthesis [10, 40]. Consequently, GDP-L-galactose formed by GME must be quickly converted to L-galactose-1-phosphate, otherwise abundant amount of GDP-L-galactose needs to accumulate in cells. Consequently, simultaneous overexpression of both *GME* and *GGP* may be suitable for strongly pulling the metabolic flux downwards and promoting rice AsA accumulation.

*GDH* and *GalLDH* are downstream genes in the L-galactose pathway. Overexpression of *GDH* in tobacco and *Arabidopsis* showed little effect on the increase in leaf AsA content [20, 21]. Our results revealed that *AtGDH* transgenic lines displayed the third best enhancement in AsA levels. Similar to our result, prior *OsGalLDH* overexpression in rice showed up to a 1.48-fold increase in AsA content [22]. However, Fukunaga et al. [15] reported that the activities of OsGDH and OsGalLDH were sufficient to convert an increased substrate to AsA. Thus, *GDH* and *GalLDH* might not be ideal candidates for co-transformation to increase AsA levels. Zhou et al. [20] reported that *GPP* overexpression in *Arabidopsis* resulted in an up to 1.5-fold increase in AsA levels compared with wild-type, and our analysis yielded similar results. Furthermore, for transgenic *Arabidopsis*, simultaneous overexpression of *GGP* and *GPP* increased leaf AsA contents up to 4.1-fold [20]. Consequently, it is suggested that the transgenic expression of both enzymes might shed light on the potential for increasing rice AsA content.

In general, plant responses to salinity by occurring in two phases, osmotic phase and ionic phase [41]. might be the best target for single according to the target genes used. nts, the test. which genes was quite similar with that In this study, overexpression of *AtGGP* or *AtGME* in rice led to improved osmotic stress tolerance. The growth rate of root and leaf of these transgenic lines were significantly higher than that in the control. The result of ion leakage analysis revealed the lesser cellular damage in transgenic lines than that in empty control. Similar results were obtained for transgenic plants overexpressing genes involved in AsA biosynthesis [2, 3, 18, 23, 42–45]. Therefore, AsA plays a key role in plants as an antioxidant in abiotic stress defense, and plants with higher AsA contents exhibited enhanced tolerance to these stresses.

Taking previous evidence and our present study together, it can be concluded that it is easy to elevate AsA levels in plants via metabolic engineering of AsA biosynthesis; however, the effects differed greatly according to the target genes used. *GGP*, *GDH* and *GME* may be the best three targets, and combining two or three target genes is a better approach for generating transgenic lines with increased AsA accumulation. Consequently, further experimental efforts are necessary to verify whether simultaneous overexpression of *GGP* and *GME*, or *GGP* and GPP would lead to a greater increase in rice AsA levels.

## Supporting Information

S1 Table PCR Primers used in this study. (DOC)

S2 Table The formula of modified Hoagland solution (pH5.5). (DOC)
