# Supplementary material for: Manipulation of the Rice L-Galactose Pathway: Evaluation of the Effects of Transgene Overexpression on Ascorbate Accumulation and Abiotic Stress Tolerance
Source: PLoS One. 2015 May 4;10(5):e0125870. doi: 10.1371/journal.pone.0125870 (PMC4418601; doi:10.1371/journal.pone.0125870)
Supplement: S1 Table — (DOC) [file pone.0125870.s001.doc]

**S1 Table. PCR Primers**

| Purpose | Gene | Sequence (5' to 3') |
| --- | --- | --- |
| cDNA amplification for vector construction | *AtGMP* | GMP-F: ATGGATCCAAGATGAAGGCACTCATTCTTGTT (*Bam*HI)  GMP-R: TAGAGCTCACATCACTATCTCTGGCT (*Sac*Ⅰ) |
|  | *AtGME* | GME-F: ATGGATCCTCAGAATGGGAACTACCAATG (*Bam*HI)  GME-R: TAGAGCTCACTCTTTTCCATCAGCCG (*Sac*Ⅰ) |
|  | *AtGPP* | GPP-F: ATGGATCCGAAAATGGCGGACAATGATTCTCT (*Bam*HI)  GPP-R:TAGGTACCTTCATGCCCCTGTAAGCCG (*Kpn*I) |
|  | *AtGDH* | GDH-F: ATGGATCCAATGACGAAAATAGAGCTTCGA (*Bam*HI)  GDH-R: TAGGTACCTTAGTTCTGATGGATTCCACT (*Kpn*I) |
|  | *AtGalLDH* | GaLDH-F: CTACTAGTCGACAAAATGCTCCGATCACT(*Spe*I+*Sal*I）  GalLDH-R: CAGGTACCTTAAGCAGTCTTGGAGACA (*Kpn*I) |
| PCR confirmation of transgenic rice | *AtGMP* | GMP-1: ATGGAGTGGTTGTTATGGAAGA  GMP-2: ATGCTTCTTGATGCGGACTC |
|  | *AtGME* | GME-1: ACGGAGGAGTTGTGTAAGCAT  GME-2: ACCATCTCAGCCATCTCATTCA |
|  | *AtGGP* | GGP-1: ACTACCAGAAGGACGATGGAGCG  GGP-2: GCAAGCAGTCAAGAACACGAGGA |
|  | *AtGPP* | GPP-1: CGCCATTGATGCCGCTAA  GPP-2: CACACGAACCACTCATCCTAA |
|  | *AtGDH* | GDH-1: ATGGAGGAACACTGTCTGAGAA  GDH-2: CCAGCAACGTCGAATCATTAAC |
|  | *AtGalLDH* | GalLDH-1: ACTTGGAGTTGTTGCTGAGGT  GalLDH-2: TGGGAGAAGGTGCTGGTATTG |
| Real-time RT-PCR | *AtGME* | GME-F1: ACGGAGGAGTTGTGTAAGCAT  GME-R1: ACCATCTCAGCCATCTCATTCA |
|  | *AtGGP* | GGP-F2: CCATCAATGTTAGTCCGATAGAGT  GGP-R2: GCCAAGTAATAAGCCTGAAAGTG |
|  | *AtGDH* | GDH-F1: CTCCTGAATGGCACCCTG  GDH-R1: CAACCAACACCGACGAAA |
|  | *OsGMP2* | OsGMP2-F: AGCCATTCTTTCTCCTCAAC  OsGMP2-R: CTCCTCCATAACCACAACAC |
|  | *OsGME1* | OsGME1-F1: AGACTTCCACTGACAGGTTTG  OsGME1-R1: TTCCAATGTTCACTGGCTCAC |
|  | *OsGME2* | OsGME2-F1: TGAATGAGATGGCTGAGATAG  OsGME2-R1: GAGCGTGTTGTCGGAGTTGC |
|  | *OsGGP* | OsGGP-F1: GCAACCATCAACCACCTCCA  OsGGP-R1: CACTATTCATTGTGCCCTCAGC |
|  | *OsGPP* | OsGPP-F: GCAACCATCAACCACCTCCA  OsGPP-R: CACTATTCATTGTGCCCTCAGC |
|  | *OsGDH* | OsGDH-F1: AAGGGGAAAAACATTACAAAG  OsGDH-R1: TTATCAATAGCGGAAGTAGACA |
|  | *OsGalLDH1* | OsGalLDH-F: AAGACGCAATCAAATCTGTGG  OsGalLDH-R: TCCTTATCCTTCGGAACCTCA |
