# Supplementary material for: Manipulation of the Rice L-Galactose Pathway: Evaluation of the Effects of Transgene Overexpression on Ascorbate Accumulation and Abiotic Stress Tolerance
Source: PLoS One. 2015 May 4;10(5):e0125870. doi: 10.1371/journal.pone.0125870 (PMC4418601; doi:10.1371/journal.pone.0125870)
Supplement: S2 Table — (DOC) [file pone.0125870.s002.doc]

**S2 Table.** **The formula of modified Hoagland solution (PH 5.5)**

| Component | Concentration (mg L^-1^) |
| --- | --- |
| **Macronutrients** | |
| NH_4_H_2_PO_4_ | 115 |
| MgSO_4_•7H_2_O | 493 |
| KNO_3_ | 606.5 |
| Ca(NO3)_2_•4H_2_O | 944.5 |
| **Micronutrients** | |
| H_3_BO_3_ | 2.86 |
| CuSO_4_•5H_2_O | 0.079 |
| MnCl_2_•4H_2_O | 1.801 |
| ZnSO_4_•7H_2_O | 0.22 |
| Na_2_MoO_4_•H_2_O | 0.03 |
| FeSO_4_•7H_2_O | 27.75 |
| EDTA-Na_2_ | 30.78 |
